# *SV2B*/miR-34a/miR-128 axis as prognostic biomarker in glioblastoma multiforme

**DOI:** 10.1038/s41598-024-55917-6

**Published:** 2024-03-19

**Authors:** D. Mustafov, S. S. Siddiqui, L. Klena, E. Karteris, M. Braoudaki

**Affiliations:** 1 School of Life and Medical Sciences, University of Hertfordshire, College Lane Campus, Hatfield, AL10 9AB UK; 2 College of Health, Medicine and Life Sciences, Brunel University London, Uxbridge, UB8 3PH UK

**Keywords:** CNS cancer, Non-coding RNAs

## Abstract

Glioblastoma (GBM) is a heterogenous primary brain tumour that is characterised with unfavourable patient prognosis. The identification of biomarkers for managing brain malignancies is of utmost importance. MicroRNAs (miRNAs) are small, non-coding RNAs implicated in cancer development. This study aimed to assess the prognostic significance of miRNAs and their gene targets in GBM. An in silico approach was employed to investigate the differentially expressed miRNAs in GBM. The most dysregulated miRNAs were identified and analysed via Sfold in association with their gene target. The candidate gene was studied via multi-omics approaches, followed by in vitro and in vivo experiments. The in silico analyses revealed that miR-128a and miR-34a were significantly downregulated within GBM. Both miRNAs displayed high binding affinity to the synaptic vesicle glycoprotein 2B (*SV2B*) 3′ untranslated region (3′UTR). SV2B exhibited upregulation within brain regions with high synaptic activity. Significantly higher SV2B levels were observed in high grade brain malignancies in comparison to their normal counterparts. SV2B expression was observed across the cytoplasm of GBM cells. Our findings underscored the downregulated expression patterns of miR-128a and miR-34a, alongside the upregulation of SV2B in GBM suggesting the importance of the *SV2B*/miR-34a/miR-128 axis as a potential prognostic approach in GBM management.

## Introduction

Glioblastoma multiforme (GBM) is an aggressive and heterogeneous primary brain malignancy characterised by its invasive nature and resistance to therapy. The tumour can originate in any part of the central nervous system (CNS), but it is frequently found in the frontal or temporal lobes^1^. GBM accounts for nearly 3350 newly diagnosed cases in the UK annually with an overall survival rate between 6–17 months^2^. Currently, aggressive multimodal therapeutic approaches involving maximal surgical resection followed by radiotherapy and chemotherapy with temozolomide have failed to improve overall survival rates and patients’ prognosis remains poor^3^. Immunotherapies incorporating treatment with nivolumab and pembrolizumab have shown no safety concerns and neurotoxicity, but no survival improvements have been noticed in recurrent GBM^4,5^. Therefore, there is an urgent demand for innovative therapies for GBM, due to its aggressive nature and the limited effectiveness of current treatment options.

Responsible for approximately 50% of all gliomas and 15% of all intracranial neoplasms, GBM is associated with a complex heterogenic molecular profile^6^. The clinical application of molecular profiles for GBM diagnosis is often limited due to the dynamic microenvironment of GBMs and their ability to transition between different molecular subtypes. Current diagnostic practices for GBM are based on magnetic resonance imaging (MRI). However, relying solely on MRI for a conclusive clinical diagnosis can lead to errors in some instances^7^. Therefore, the need for tissue biopsy in the diagnostic decision-making process is nearly inevitable. Nonetheless, this approach has its limitations, including the invasive nature of the procedure, the lack of serial monitoring of the malignancy, tissue heterogeneity and the potential risk of neurological damage. To address these challenges, we have turned our attention to microRNAs (miRNAs).

MiRNAs are a class of small non-coding, single-stranded RNA molecules widely found in eukaryotic organisms consisting of approximately 22 nucleotides. The process of miRNA biogenesis undergoes intricate regulation at both the transcriptional and post-transcriptional levels to produce fully functional, mature miRNAs, which play a vital role in maintaining cellular homeostasis^8^. However, alternations of miRNAs functional properties do occur occasionally and could potentially lead to a transcriptome imbalance associated with different malignancies^9^. Deregulation of miRNAs in GBM can result from various faulty events during their biogenesis, such as amplifications, deletions, epigenetic modifications, translocations, and silencing. The overexpression of certain miRNAs can lead to the suppression of tumour suppressor genes, while the downregulation of others can be associated with increased expression of oncogenes. Both scenarios could affect cell proliferation, differentiation, and apoptosis, ultimately contributing to the growth and progression of tumours^10^. The detection of miRNA signatures in primary brain tumours has provided valuable insights into the diagnosis, prognosis, and monitoring of patients^11^, but more research is necessitated to unravel their exact role in the progression of those types of tumours.

Herein, we aimed to screen for the differentially expressed miRNAs and their target genes within GBM patients to identify valid prognostic biomarkers or therapeutic targets. Specifically, in silico analysis revealed that miR-34a and miR-128a were significantly downregulated in GBM cells and patients verifying their tumour suppressor properties. Additional bioinformatics analysis demonstrated that a common target gene for both miRNAs was the synaptic vesicle glycoprotein 2B (*SV2B*) gene which was found upregulated in GBM patients and hence manifesting oncogenic properties. The effect of current miRNA regulation on SV2B protein function was validated using brain tumour patient samples, including GBM. Based on current findings, we are proposing that the *SV2B*/miR-34a/miR-128 axis could serve as a putative prognostic biomarker for glioblastoma multiforme.

## Results

### miRNA analyses

**Figure 1:**
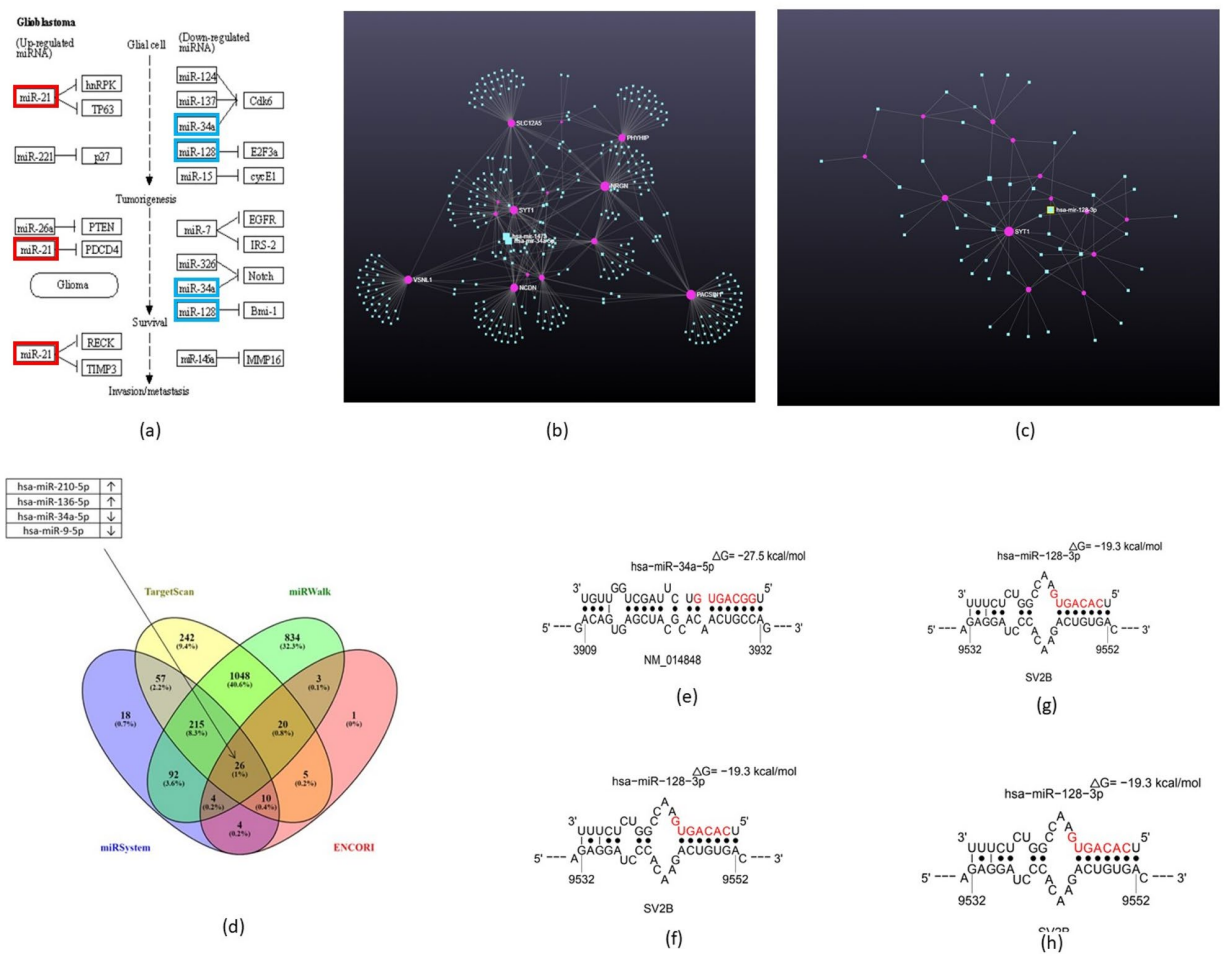
miRNA-*SV2B* interactions. (**a**) KEGG pathway analysis depicted the most differentially expressed miRNAs profiles in 529 GBM cases (grade 4). miR-21 and miR-221 (red boxes) were seen to be upregulated, whereas miR-34a and miR-128 (blue boxes) were downregulated. (**b**) miR-34a was depicted as one of the most important miRNAs involved in the overall gene regulation for *SV2B* co-expressed genes (*HRNBP3, SULT4A1, SLC12A5, PACSIN1, GRIN1, VSNL1, PHYHIP, SYN2, CAMK2A, GLS2, NCDN, NRGN, SYT1, AK5, NAPB*). (**c**) miR-128a was observed to be one of the most important brain specific miRNAs regarding gene hub associated with *SV2B.* (**d**) miR-34a-5p was identified as downregulated and depicted as a main target for *SV2B* out of 26 common miRNA gene targets. (**e**) 3′UTR of *SV2B* and miR-34a binding at site position 3909–3932, ΔGhybrid = − 27.500 kcal/mol. (**f**) 3′UTR of *SV2B* and miR-128-3p binding at site position 8986–8999, ΔGhybrid = − 17.900 kcal/mol. (**g**) 3′UTR of *SV2B* and miR-128-3p binding at site position 9532–9552, ΔGhybrid = − 19.300 kcal/mol. (**h**) 3′UTR of *SV2B* and miR-128-3p binding at site position 5671–5685, ΔGhybrid = − 17.600 kcal/mol.

Throughout our bioinformatics analysis, illustrated in Table 1, we analysed miRNAs and their gene targets using a formatted miRNA summary report, obtained from the NCBI and miRSystem databases. The summary of the hits between miRNAs and their target genes allowed us to select the four miRNAs with evident relevance within GBM pathogenesis, including miR-21-5p, miR-34a, miR-128a-3p, and miR-221-3p. Of these, miR-34a showed the highest number of hits against GBM oncogenes. Subsequent KEGG pathway analysis aided in depicting the differential expression profiles of these miRNAs (Fig. 1a). miR-34a and miR-128a were shown to be downregulated in GBM, whilst miR-21a and miR-221-3p were seen to be upregulated. Further analysis of the commonly co-expressed genes with *SV2B* and their miRNA regulatory pathways depicted miR-34a as the most important miRNA involved in overall gene regulation for this hub, whereas miR-128a was seen as the most important brain specific miRNA regarding the regulation of the gene hub and *SV2B* (Fig. 1b,c). Furthermore, common miRNAs targeting the *SV2B* gene including miR-128a and miR-34a were assessed by employing four databases: miRSystem, TargetScan, miRWalk, and ENCORI. The findings indicated that there were 26 common miRNA gene targets (1%) that were identified by all four databases. Within this set of shared miRNAs, miR-34a-5p was seen as downregulated and depicted as the main target for the gene (Fig. 1d). We selected the downregulated miR-34a and miR-128a for subsequent Sfold base-pair matching analysis. As illustrated in Fig. 1e, miR-34a featured a well conserved binding site for the 3′UTR of *SV2B* at site position 3909–3932, with a ΔGhybrid value of − 27.500. The binding type was 7mer-A1. Figure 1f–h revealed the Sfold predicted binding sites between *SV2B* and miR-128a. The outcome of the 3′UTR-miRNA binding depicted three site positions, including 8986–8999, 9532–9552, and 5671–5685. The hybrid binding interaction at these sites was − 17.900, − 19.300, and − 17.600, respectively. The seed interactions were described as 7mre-A1 for the first two sites, and as 6mer for the latest.

**Table 1:** Identification of miRNAs and associated target genes.

Name of miRNAs	Gene name and total no. of hits							
	*CDK6*	*NOTCH1*	*NOTCH2*	*JAG1*	*TIMP3*	*E2F3*	*BMI1*	Total
hsa-miR-21-5p	5	0	0	7	7	3	0	22
hsa-miR-34a-5p	7	7	8	8	0	8	0	38
hsa-miR-128a-3p	1	0	0	4	0	8	7	21
hsa-miR-221-3p	3	0	0	1	7	0	6	17

The four depicted miRNAs; hsa-miR-21-5p, hsa-miR-34a-5p, hsa-miR-128-3p, hsa-miR-221-3p, aligned against the oncogenes Cyclin-dependent kinase 6 (*CDK6*), Neurogenic locus notch homolog protein 1 and 2 (*NOTCH1* and *NOTCH2*), Jagged Canonical Notch Ligand 1 (*JAG1*), TIMP Metallopeptidase Inhibitor 3 (*TIPM3*), E2F Transcription Factor (*E2F3*), and B lymphoma Mo-MLV insertion region 1 homolog (*BMI1*) found in GBM.

### SV2B expression

**Figure 2:**
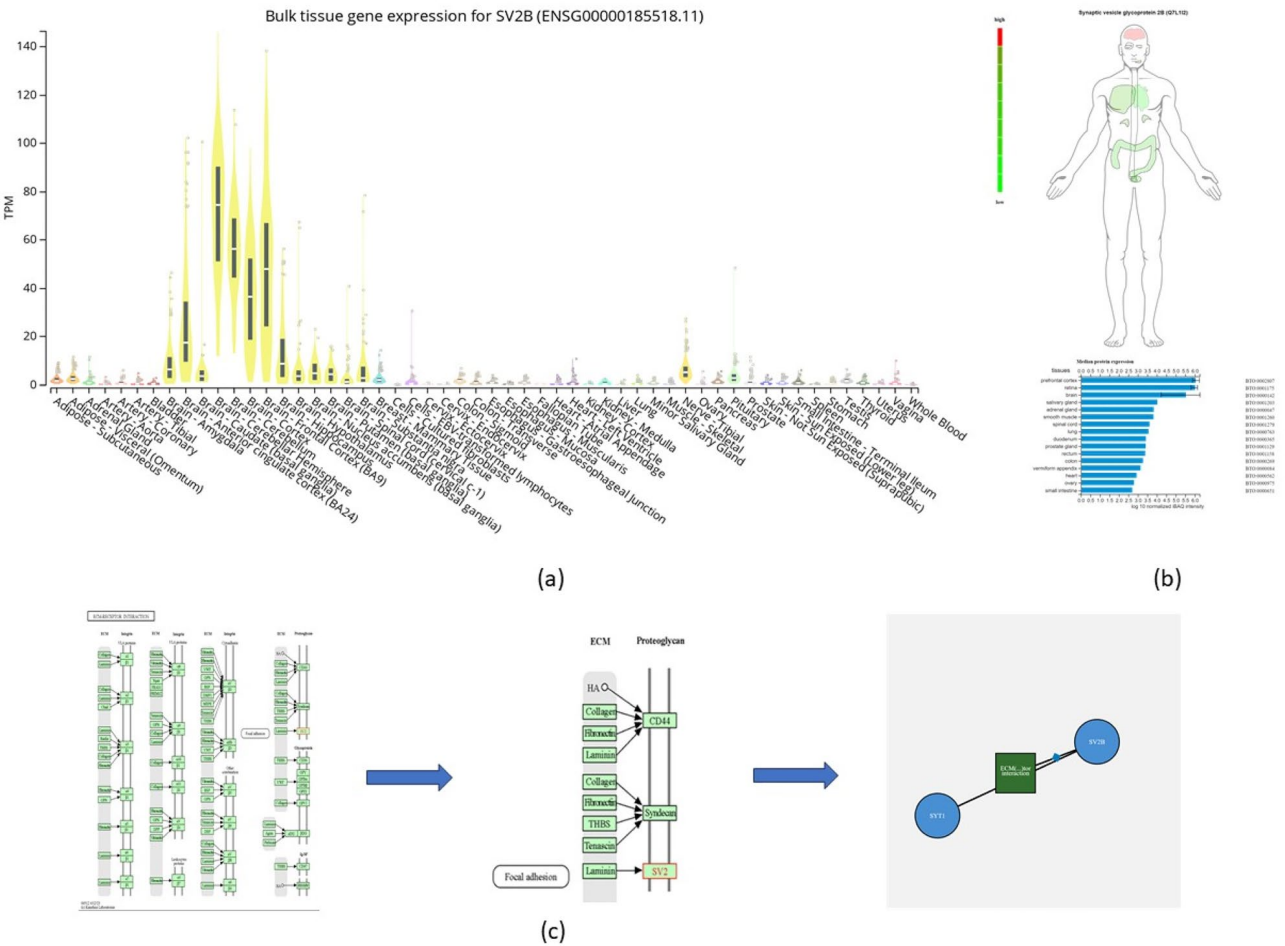
Expression of SV2B across the human body and its relation to biological pathways. (**a**) *SV2B* expression was observed to be highly elevated in various brain regions, including the frontal cortex (BA9) (8.9 times) (n = 56 females; n = 153 males), cortex (8.2 times) (n = 74 females; males = 181), cerebellar hemisphere (7.2 times) (n = 58 females; n = 157 males), anterior cingulate cortex (BA24) (7.1 times) (n = 48 females; n = 128 males), and cerebellum (5.8 times) (n = 67 females; n = 174 males). (**b**) SV2B proteomics analysis depicted that the protein’s highest abundance was within the prefrontal cortex (6.01 iBAQ), the retina (5.98 iBAQ), and the brain as a whole (5.48 iBAQ). (**c**) The SV2 protein family was seen to be involved in extracellular matrix–receptor interactions (ECM) with laminins. *SV2B* was seen to interact closely with *SYT1* as part of the ECM-receptor interaction signalling.

Figure 2a illustrates the normal expression of the *SV2B* gene within various human tissues. The results demonstrated that the expression of this gene is highly elevated in the following brain regions: frontal cortex (BA9) (8.9 times), cortex (8.2 times), cerebellar hemisphere (7.2 times), anterior cingulate cortex (BA24) (7.1 times), and cerebellum (5.8 times). Furthermore, the proteomics analysis shown in Fig. 2b revealed that the median protein log^10^ normalized iBAQ intensity expression of SV2B was highest within the prefrontal cortex, the retina, and the brain as a whole (6.01, 5.98, and 5.48 iBAQ, respectively). The incorporated KEGG pathway analysis as shown in Fig. 2c depicted that members of the SV2 protein family were classed as proteoglycans that were involved in extracellular matrix–receptor interactions (ECM) with laminins. Specifically, *SV2B* was seen to interact closely with *SYT1* as part of the ECM-receptor interaction signalling.

### String and survival analyses

**Figure 3:**
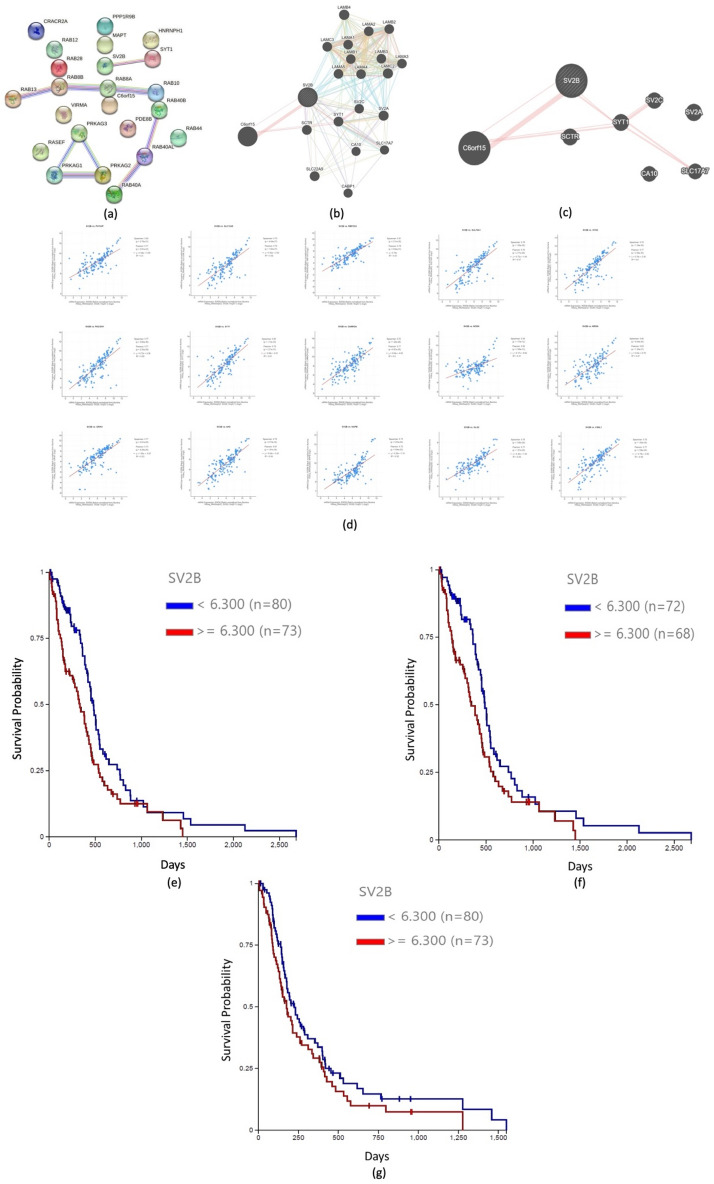
*SV2B* string interaction network. (**a**) String interaction analysis of *SV2B* depicted that synaptotagmin 1 (*SYT1*) was directly co-expressed alongside *SV2B* (score 0.128). (**b**,**c**) GeneMANIA interactive network also illustrated strong co-expression between *SV2B* and *SYT1*, *C6orf15, SCTR, SV2C,* and *SLC17A7*. (**d**) *HRNBP3, SULT4A1, SLC12A5, PACSIN1, GRIN1, VSNL1, PHYHIP, SYN2, CAMK2A, GLS2, NCDN, NRGN, SYT1, AK5,* and *NAPB* positively co-expressed with *SV2B*. (**e**) Survival analysis depicted that higher levels of *SV2B* were associated with significantly shorter overall survival (p < 0.01119). (**f**) Disease specific survival analysis indicated that high *SV2B* expression was associated with shorter disease specific survival (p < 0.02197). (**g**) Progression free survival GBM analysis did not depict statistically significant differences.

Figure 3a illustrates a string interaction analysis of the *SV2B* gene and any co-expressed genes. As observed, synaptotagmin 1 (*SYT1*) was directly co-expressed alongside *SV2B* (score 0.128). Meanwhile, experimental, and biochemical data have demonstrated the relation between these two genes (score 0.292). Furthermore, a GeneMANIA interactive network was constructed, which also confirmed the strong co-expression between *SV2B* and *SYT1*, alongside *C6orf15, SCTR, SV2C,* and *SLC17A7*. The latest also showed that SV2B interacted with a great majority of laminins, supporting our aforementioned KEGG pathway observations (Fig. 3b,c). Our analysis also depicted the 15 genes that most positively correlated and co-expressed alongside *SV2B*, including: *HRNBP3, SULT4A1, SLC12A5, PACSIN1, GRIN1, VSNL1, PHYHIP, SYN2, CAMK2A, GLS2, NCDN, NRGN, SYT1, AK5,* and *NAPB* (Fig. 3d). Furthermore, survival analysis of primary GBM samples demonstrated that patient groups expressing higher levels of *SV2B* in comparison to those with lower expression levels had significantly shorter overall survival and disease specific survival (p < 0.01119 and p < 0.02197, respectively) (Fig. 3e,f). The variations between *SV2B* expression profiles within the progression free survival GBM analysis did not depict statistically significant differences (Fig. 3g).

### Immunohistochemistry SV2B

**Figure 4:**
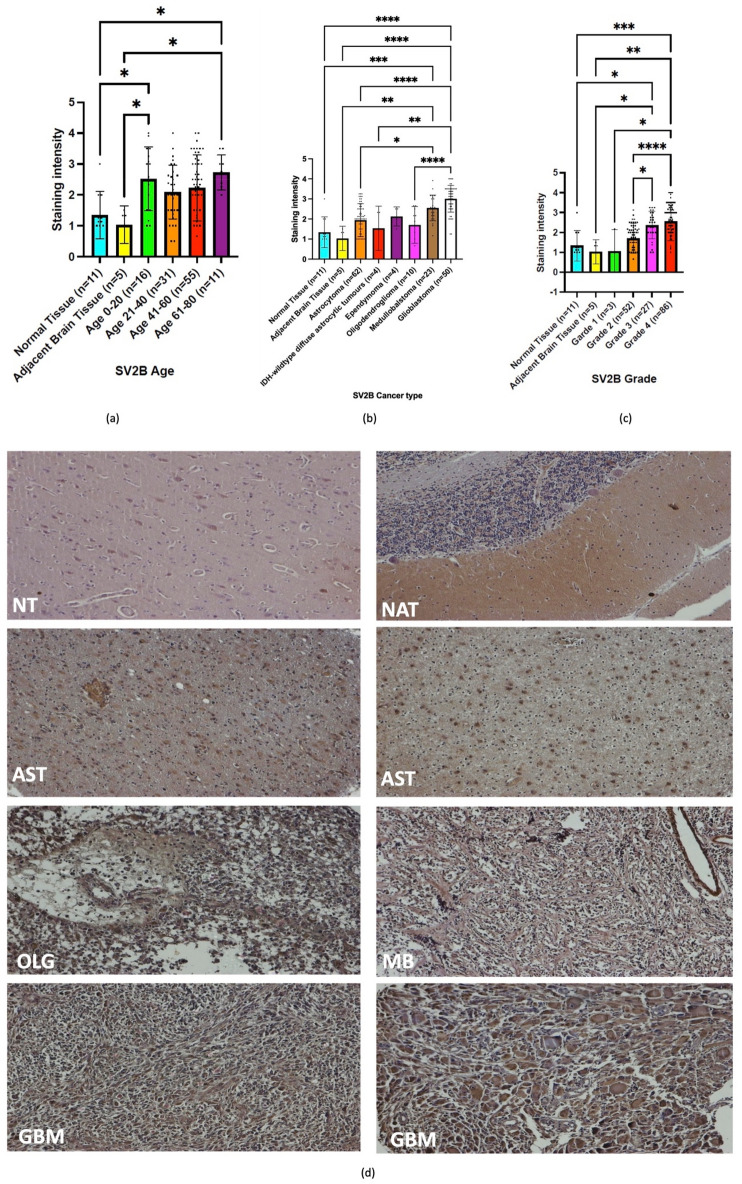

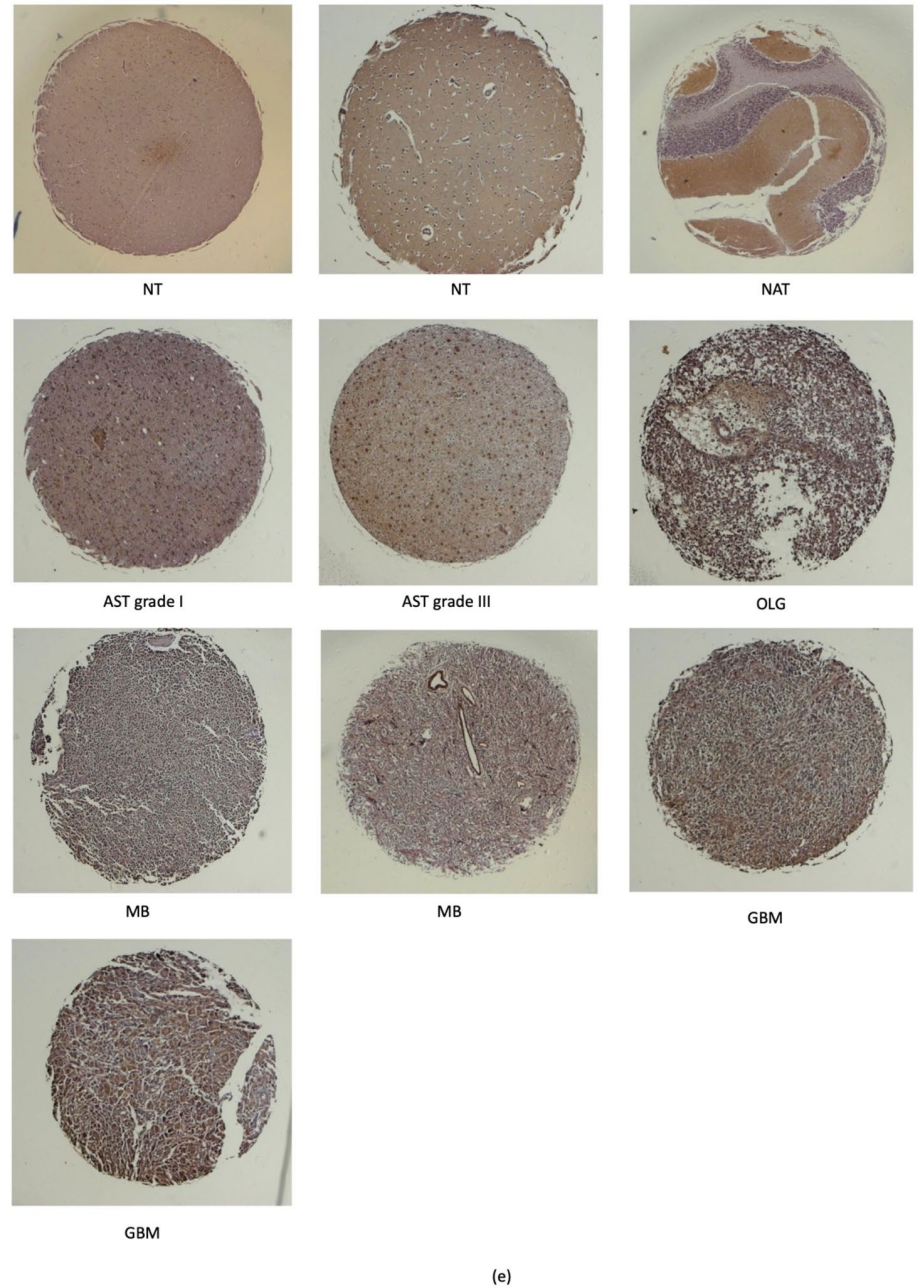
Immunohistochemistry analyses of SV2B. (**a**) The age groups of 0–20 years and 61–80 years reached significant difference in expression of SV2B when compared to their normal counterparts (Normal Tissue versus Age 0–20, p < 0.0249; Adjacent Brain Tissue versus Age 0–20, p < 0.0344; Normal Tissue versus Age 61–80, p < 0.0200; Adjacent Brain Tissue versus Age 61–80, p < 0.0231). (**b**) Significantly higher levels of the protein were observed within glioblastomas when compared to astrocytoma, IDH-wildtype diffuse astrocytic tumors, and oligodendrogliomas (p ≪ 0.0001, p < 0.0056, and p ≪ 0.0001, respectively). Medulloblastoma samples also reached statistical significance of high abundance of SV2B in comparison to astrocytoma (p < 0.0245). (**c**) Significant differences between tumor grades 3 and 4 in comparison to normal brain tissue were observed (p < 0.0133 and p < 0.0002, respectively). Significant differences between grades 1 and 2 in comparison to grade 4, and grades 2 and 3 we also depicted (Grade 1 versus Grade 4, p < 0.0390; Grade 2 versus Grade 4, p < 0.0001; Grade 2 versus Grade 3, p < 0.0184). (**d**) Microscopic images of SV2B stained tissues sections illustrating the staining intensity within normal brain tissue, tumor adjacent tissue, and different brain malignancies at × 100 magnification. (**e**) Microscopic images of SV2B stained tissues cores of normal brain tissue, tumor adjacent tissue, and different brain malignancies at × 40 magnification. *NT* normal tissue, *NAT* normal adjacent tissue, *AST* astrocytoma, *OLG* oligodendroglioma, *MB* medulloblastoma, *GBM* glioblastoma, *Ns* non-significant, *p < 0.05, **p < 0.01, ***p < 0.001, ****p < 0.0001.

The immunohistochemical analysis of SV2B performed within patient cohorts and normal brain samples revealed higher expression of the protein in all age patient groups in comparison to normal and normal adjacent brain tissue samples. However, only the age groups of 0–20 years and 61–80 years reached a significant difference regarding protein expression when compared to its normal counterparts (Fig. 4a). Figure 4b illustrates the different patterns of SV2B expression within different tumour types. The analysis depicted significant differences of SV2B levels between astrocytomas, IDH-wildtype diffuse astrocytic tumours, and oligodendrogliomas when compared to glioblastomas, the latter expressing higher protein abundance within patient samples. Medulloblastomas also possessed a significantly higher abundance of SV2B in comparison to astrocytomas. Malignant brain tumours evidently expressed higher levels of SV2B when compared to normal and normal adjacent tissue samples, reaching significance. Figure 4c depicted a significant increase of SV2B expression with increasing tumour grade. Significant differences of measured SV2B levels were seen between grades 1 and 2 in comparison to grade 4 brain tumour samples. Furthermore, the differences between grades 2 and 3 we also denoted with significance. Grades 3 and 4 were shown to express much higher levels of SV2B in comparison to lower tumour grades. As illustrated in the panel of microscopic images in Fig. 4d, the intensity of the brown colourised tissues sections increased within high grade tumours. The panels in Fig. 4e showed different patient samples with progressing tumour stages.

### Immunofluorescence

**Figure 5:**
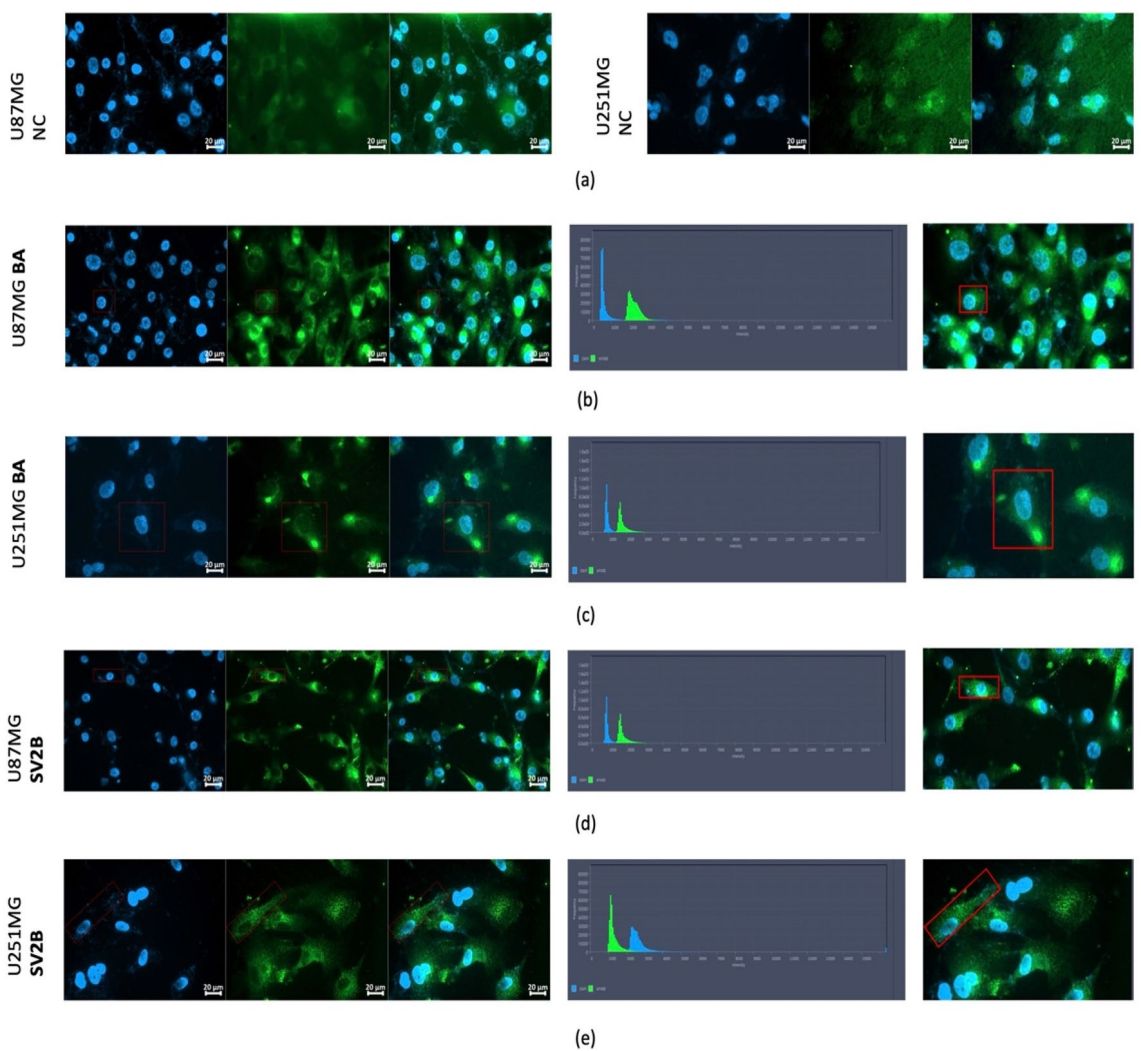
SV2B Immunofluorescence analysis. (**a**) There was no specific binding observed in the negative condition of the experiment. (**b**) Uniform staining across U87MG cells with beta-actin (positive control). (**c**) Uniform staining across U251MG cells with beta-actin (positive control). (**d**) The U87MG cell line exhibited intense SV2B staining (~ 1300–2200 range) enriched around the nucleus. (**e**) U251MG cells exhibited lower intensity of staining for SV2B (~ 900–2000). All microscopic images were taken under × 40 magnification.

The immunofluorescence analyses of stained GBM cells revealed that there was a specific localisation of SV2B within the cytoplasm of GBM cells. Figures 5a illustrated the negative control condition which did not visualise any specific binding. Βeta-actin was used as a positive control and showed similarly uniform staining across both the U87MG and U251MG cell lines (Fig. 5b,c). Figure 5d,e represented that SV2B was enriched around the nucleus, suggesting a possibility of being localised in the endoplasmic reticulum or Golgi. Staining intensity was variable across the two cell lines. The U87MG cell line exhibited more intense SV2B staining (~ 1300–2200 range) compared to the U251MG cell line which exhibited lower intensity of staining for SV2B (~ 900–2000). Despite the higher intensity, there were less SV2B U87MG positive cells and therefore lower signal frequency than in U251MG cells, where the signal intensity was lower, but staining frequency was higher.

## Discussion

The limited success in achieving favourable clinical outcomes for primary brain tumours has spurred increased efforts to comprehend the role of miRNAs. Our research attempted to elucidate the role of miR-128a and miR-34a, as emerged through our analyses, and their action upon their target gene *SV2B*.

The conducted in silico analysis demonstrated that miR-21 and miR-221 were overexpressed in GBM tumours, whereas miR-128a and miR-34a were downregulated. This was further supported by the KEGG pathway analysis, where miR-21 and miR-221 were observed to be upregulated, whilst miR-34a and miR-128a were found to be downregulated. Given the oncogenic nature of miR-21 and miR-221, they were excluded from further analysis in relation to *SV2B.*

The potential therapeutic use of miR-34a, which might function as a tumour suppressor by targeting gene promoters within the p53 pathway has arisen for patients with GBM^12^. Vaitkiene et al., 2019 also demonstrated lower miR-34a expression which was associated with larger tumour volume, decreased physical functioning, and lower Karnofsky Performance Status (KPS) scores in patients with GBM. However, these findings were described as preliminary, due to the small sample size incorporated in that study. miR-34a was also found significantly downregulated within patients’ serums samples highlighting its relevance as a promising non-invasive biomarker for GBM^13^. However, larger sample size validations are further required to strengthen its clinical importance.

The miR-128a is an intronic miRNA primarily located in the brain and has been previously associated with normal brain development^14^. However, miRNA assays, RT-qPCR, and western blot analyses have indicated that miR-128a is expressed at lower levels in aggressive solid brain tumours, including GBM. On the contrary, Roth, et al., 2011 demonstrated that there were elevated levels of miR-128a within blood samples^15^. Our *in-silico* analysis revealed that miR-128a was downregulated within glioblastoma patient samples suggesting its tumour suppressive properties. These findings were in line with previous bioinformatics analyses which had identified low miR-128 levels in serum and tissue patient samples^16^. The low levels if miR-128a were associated with high pathological grade and low Karnofsky Performance Status score (KPS). This in turn has suggested that miR-128 could act as a serum detectable biomarker in GBM patients.

Our results demonstrated a potential interaction between miRNAs and the *SV2B* gene. *SV2B* is involved in synaptic vesicle trafficking and neurotransmitter release^17^. Our findings showed that miR-128a and miR-34a featured conserved binding sites towards *SV2B*. However, miR-34a stood out as a potential target for *SV2B*, as detected in all four miRNA-databases. To the best of our knowledge, the potential prognostic value of the miR-34a/mir-128a/*SV2B* axis has not been reported before. Previous work has demonstrated that miR-330-3p was implicated in *SV2B* regulation via directly targeting *SV2B* mRNA and inhibiting its expression in pancreatic β-cells^18^. In glioma tissues and cells, miR-330-3p was observed to exert low expression levels compared to adjacent tissues and normal astrocytes^19^. Our *in-silico* analyses depicted that miR-330-3p and miR-34a targeted *SV2B* (Supplementary Table 1). Thus, an association between the two miRNAs and their target can potentially occur leading to the degradation of the gene. According to Morris et al., 2019 mir-134 was found to target *SV2B* in the brain^20^. In line with these findings, our bioinformatic analysis demonstrated that miR-134 had 167 binding sites in the 3′UTR-seedless region of *SV2B* (Supplementary Table 2) suggesting its high binding affinity to *SV2B*. In neuronal cells, miR-134 can downregulate *SV2B* expression, potentially affecting synaptic transmission and plasticity. The brain regional distribution between *SV2B* and miR-134 led to the suggestion that other brain specific miRNAs, such as miR-128a could act as targets for *SV2B* and thus influence its expression.

Expressed as a major form of the SV2 family, *SV2B* distribution across the brain was previously depicted and the gene was found to be exclusively expressed within glutamatergic neurons^21^. An immunohistochemical study has found the expression of the synaptic protein within the cerebellum, basal ganglia, and hippocampus^22^. This was in support to our findings which demonstrated that *SV2B* was predominantly expressed within synaptically active brain areas, such as the frontal cortex, cortex, cerebellar hemisphere, anterior cingulate cortex, and cerebellum. Given the primary function of *SV2B*, the protein product acts as a store of neurotransmitters and releases them upon neuronal excitation^23,24^. This has suggested that genes that align with the spatial location of GBM were associated with neuroactive ligand–receptor interactions and biological processes including the regulation of synapse organization, the modulation of chemical synaptic transmission, and interactions involving neuroactive ligands and receptors. Furthermore, genes associated with synapses, which possess postsynaptic structures and glutamate receptors, such as *SV2B*, have been observed to exhibit elevated expression levels in various subgroups of malignant gliomas^25^. When exposed to high levels of Ca^2+^ glioma cells increase the release of glutamate into the tumour microenvironment (TME). This in turn leads to excessive glutamatergic and calcium ion signalling and promotes the spread of glioma calls^26^. Our results demonstrated that high levels of *SV2B* led to shorter overall survival in patients with GBM, suggesting its diagnostic value for GBM patient cohorts. The energy conductivity observed in gliomas may indicate an intensified information transmission. Conversely, it is worth noting that this abnormal and prolonged activity can result in a reduction in the physiologically normal conductivity. Despite the heightened activity in gliomas, it is important to acknowledge that their activity may not be solely dependent on *SV2B*.

Our STRING analysis indicated that *SV2B* is significantly co-expressed with genes involved in the ECM-receptor interaction, making it an important hub gene associated with signalling transduction. Previous evidence suggested that *PAK-1* and *SV2B* might act as GBM prognostic biomarkers when studied together^27^. As apparent from our KEGG pathway and GeneMania results, *SV2B* had a very strong interaction and co-expression with Synaptotagmin-1 (*SYT1*) instead. *SYT1* is situated on the vesicular membrane of nerve and endocrine cells. It acts as a primary calcium (Ca^2+^) sensor in the mechanisms of neurotransmission and hormone secretion, and it plays a crucial role in processes triggered by Ca^2+^ for secretion^28^. Given the involvement of *SYT1* and its calcium-binding protein product, this gene has appeared in GBM hub oncogene nodes alongside *SV2B*^29,30^. Moreover, a larger scale analysis of glioblastoma and normal brain tissue samples obtained from the TCGA and GEO databases revealed that *SYT1* was one of the core genes associated with GBM progression among the 552 differentially expressed genes in that analysis^31^. Nevertheless, Losada-Pérez et al.^30^ identified that *SYT1* gathers on the membrane of *Drosophila* glial cells, which in turn appears to intensify its expression within GBM samples. These statements, along with our findings suggested that the *SV2B* and *SYT1* could act as potential prognostic biomarkers in combination for GBM patients as both oncogenes are associated with shorter overall survival.

Previous bioinformatics studies suggested that *SV2B* expression may be associated with glioma grade or unfavourable prognosis, suggesting its oncogenic properties^32,33^. Additionally, *SV2B* was found to be overexpressed within breast cancer tissue when compared to normal tissue^34^. This is in support with our IHC results, which depicted significantly high SV2B protein expression in high grade brain malignancies, such as medulloblastoma and GBM, respectively. To the best of our knowledge, no current immunohistochemical evidence of expression for SV2B within patient cohorts diagnosed with brain tumours exists. One of the possible reasons for the high accumulation of SV2B within high grade brain malignancies could be widely distributed *SV2B* messenger neurotransmitter within the brain. In turn, that leads to elevated intracellular Ca^2+^ levels which stimulate cell proliferation and migration, leading to glioma cells acquiring their tumorigenic characteristics^35^. Additionally, SV2B has been demonstrated to be associated with energy metabolism, as the substantial energy requirements within the tumour may heavily depend on SV2B for regulating transporter protein activity, glucose transport, and other functions^36^. Our IHC findings revealed that the expression of SV2B increased with tumour grade, which suggested that highly metabolically active malignancies might require more of the protein to be able to proliferate and metastasise. GBMs and medulloblastoma represent advanced stage in the development of brain malignancies, where numerous genes are differentially expressed to varying extents. Additionally, half of the newly diagnosed high grade brain tumours are found in patients over 65 years^37^. Our results showed that patients in the age group of 61–80 years presented significantly high expression of SV2B in comparison to their normal counterparts suggesting its prognostic relevance in high grade brain malignancies. The significant overexpression of SV2B in the age groups of 0–20 years also suggested that the protein is highly expressed within paediatric brain malignancies too. Moreover, the elevated levels of SV2B found in highly malignant paediatric brain malignancy cases could be associated with their expression in brain regions characterized by pronounced synaptic plasticity. Furthermore, the observed difference between NAT and high-grade tumours is worth noting. The significant under expression of SV2B within NAT could be a result of the particular characteristics within this type of tissue that makes it a separate entity and distinguishes it from both healthy and tumour tissue. Thus, it could be suggested that *SV2B* could potentially act as an oncogene hence its high abundance in highly malignant brain tumours. Furthermore, the expression levels SV2B suggested that it can act as a prognostic marker, enhancing its clinical relevance in combating yet incurable highly malignant brain tumours, such as glioblastoma and medulloblastoma. Therefore, SV2B holds promising hopes in the search for new biomarkers. However, there is still little known regarding their potential oncogenic mechanisms within brain cancers.

In summary, this study focused on the role of miRNAs in brain tumours, particularly in GBMs. Our research aimed to unveil the expression patterns of miR-34a and miR-128a exploring their potential as prognostic biomarkers and therapeutic targets. In silico analyses revealed downregulation of miR-128a and miR-34a in GBM suggesting their tumour suppressor roles. Also, the study investigated the interaction of miR-34a and/or miR-128a and *SV2B,* a putative target gene. SV2B was found upregulated in high grade glioma tissues and exerted potential as a clinical prognostic marker.

Nevertheless, it is worth noting the potential limitations associated with this study, including the genetic and molecular variations between paediatric and adult brain malignancies, as well as the differences in their treatment approaches. Additionally, sample size limitations in patient and control groups must not be disregarded. The inclusion of functional assays could strengthen the reliability of our conclusions and could guide future experimental investigations. Nonetheless, our research could still pave the way for clinical interventions aimed at miR-128a and miR-34a and their target gene, *SV2B.*

## Methods

### In silico miRNA analyses

Bioinformatic analysis was conducted to select the miRNAs with potential clinical relevance for the diagnosis, prognosis, and treatment of glioblastoma. The NCBI database was incorporated in the identification of GBM dysregulated genes by employing the following filter form category “Popular”: Entrez Gene records that are current; gene records annotated on RefSeq chromosome or contig accessions; gene records associated with gene-specific reports in dbSNP (VAR); and Genes with records in the ClinVar database. The miRSystem database (http://mirsystem.cgm.ntu.edu.tw/index.php) was used to generate a report of miRNAs targeting the GBM dysregulated genes from the NCBI report. The default parameter in miRSystem was set at three algorithm hits in order to increase the reliability of the generated results. The Kyoto Encyclopaedia of Genes and Genomes (KEGG; https://www.genome.jp/kegg/pathway.html)^38^ was consequently utilised to study differentially expressed miRNAs and genes associated with glioblastoma and their relevance in tumorigenesis. The sample size included 529 GBM cases (grade 4). The miRBase database (https://mirbase.org) was used for miRNA sequences and annotations, whereas the Software for Statistical folding of nucleic acids and studies of regulatory RNAs (Sfold) (https://sfold.wadsworth.org/cgi-bin/starmirWeb.pl)^39^ provided the probable binding sites of the chosen to miRNAs to their gene target. Subsequently, a Venn diagram of common target miRNAs for *SV2B*, was created via Venny2.1.0 (https://bioinfogp.cnb.csic.es/tools/venny/). Four different miRNA tools were employed to generate miRNAs that target *SV2B*. These included: miRSystem, miRWalk (*SV2B*—XM_017022761) (http://mirwalk.umm.uni-heidelberg.de/), TargetScan (*SV2B*-ENST00000394232.1), and ENCORI (The Encyclopaedia of RNA Interactomes) (https://starbase.sysu.edu.cn/). The four databases were employed to provide stronger evidence for functional miR–mRNA target interactions (MTIs) and to reduce the likelihood of false positive results. Following that, the miRNA-centric network visual analytics platform miRNet2.0 (https://www.mirnet.ca/)^40^ was important in building a network of the miRNA-target interactions between the commonly co-expressed genes with *SV2B*.

### In silico target gene analysis

To study the tissue gene expression of *SV2B* (ENSG00000185518.11), the Gtex Bulk database (https://www.gtexportal.org/home/gene/SV2B, GTEx Analysis Release V8, dbGaP Accession phs000424.v8.p2) was used. Expression values were shown in transcripts per million. Box plots were illustrated as median and 25th and 75th percentiles. Any points above or below the 1.5 times interquartile range were displayed as outliers. The protein expression of SV2B was depicted via the ProteomicsDB database (proteomics data—MS1, Q7L1I2) with no gender comparison conducted (https://www.proteomicsdb.org/proteomicsdb/#human/proteinDetails/Q7L1I2/expression). To elucidate the indolent of SV2B in a given biological pathway, KEGG Pathway (has:9899) and ProteomicsDB Pathway (Q7L1I2) analyses were conducted (https://www.genome.jp/entry/hsa:9899; https://www.proteomicsdb.org/proteomicsdb/#human/proteinDetails/Q7L1I2/interactions). To assess the effects of differential expression of SV2B upon GBM survival states, the Xena Functional Genomics Explorer (https://xenabrowser.net/)^41^ was used where the TCGA Glioblastoma study (631 GBM patient samples, grade 4) was selected for analysis. Recurrent GBM tumour samples were omitted from the final analysis reducing the sample size to 619 primary GBM tumour samples (grade 4; aged 10–89 years old; n = 230 females; n = 389 males). String interaction analysis for *SV2B* and its co-expressed genes was conducted by GeneMANIA (https://genemania.org/search/homo-sapiens/sv2b/)^42^ and STRING (https://string-db.org/cgi/network?pollingId=bsDyNqzn53bk&sessionId=bLTkmworrpXG&urldisam=b7Gi9w2abdot).

### Cell culture

The glioblastoma cell lines U251MG and U87MG were obtained from the American Type Culture Collection (ATCC, Manassas, VA, USA). U251MG and U87MG cells were cultured in complete minimum essential media (MEM) (GibcoTM, Bleiswijk, NL) supplemented with 10% FBS (Gibco, Bleiswijk, NL) and 1% Pen/Strep (Gibco, Bleiswijk, NL). Maintained in a humidifying incubator at 37 °C with 5% CO_2_, the cells were regularly tested for mycoplasma contamination.

### Immunohistochemistry

Paraffin-embedded, commercially available, brain tumour tissue microarray slides (US Biomax Inc., London, UK; GL631, GL803c, GL2082a) were obtained in compliance with Health Insurance Portability and Accountability Act (HIPAA) approved protocols to ensure ethical standards. The slides contained 351 biopsy cores from 207 patients. The slides featured different brain malignancy types at different stages including astrocytoma, oligoastrocytomas (referred to as IDH-wildtype diffuse astrocytic tumours across the manuscript according to the world health organisation (WHO) classification of brain tumours, 2021), ependymomas, oligodendrogliomas, medulloblastomas, and glioblastomas, alongside normal and normal adjacent control tissue (Supplementary Table 3). The term “oligoastrocytoma” occurring in the IHC tissue microarray was used according to the old WHO classification brain tumours. This has been replaced with the term “IDH-wild type astrocytic tumours” according to the 2021 WHO classification of brain tumours which was used across this manuscript^43^. The protocol followed was previously described by Filipe et al.^44^. Post deparaffinized, rehydrated, antigen retrieval, treatment with H_2_O_2_, and blocking, the slides were incubated overnight at 4 °C with the SV2B primary antibody (1:200 dilution) (ProteinTech, Manchester, UK). Following an overnight incubation at 4 °C, the slides were incubated with a secondary anti-rabbit antibody from the Zytochem Plus HRP-DAB Kit (HRP008DAB-RB, Zytomed Systems, UK) for 1 h. Then, streptavidin-HRP conjugate was added, followed by a 45min incubation. Staining and counterstaining with DAB and haematoxylin was performed, respectively. Dehydration was performed using ethanol and HistoClear, and coverslips were applied with DPX mounting medium. Immunoreactivity was assessed by three independent viewers using a light microscope (Zeiss Microscopy, Oberkochen, Germany). The extent of brown staining was scored based on the percentage of positively stained cells. Scoring categories included: 0 (< 5% cells), 1 (5–25% cells), 2 (26–50% cells), 3 (51–75% cells), and 4 (> 75% cells). The average score from the three independent assessments was calculated.

### Immunofluorescence

U87MG and U251MG glioblastoma cells were seeded on round coverslips in a 12-well plate at a cell density of 2.5 × 10^5^ cells/well. The cells were grown in their respective media type for 24 h. Following that, the cells were fixed with 4% paraformaldehyde (PFA) (Sigma-AldrichTM, Dorset, UK) for 45 min. Two 5-min wash steps with phosphate buffered saline (PBS) (ThermofisherTM, CA, USA) were then performed. Blocking of the slides with 5% bovine serum albumin (BSA) (Sigma-AldrichTM, Dorset, UK) for 1 h was then carried out. The blocking solution was aspirated, and cells were incubated in primary antibody (in 5% BSA) overnight at 4 °C with gentle agitation. The dilutions for the SV2B (ProteinTech, Manchester, UK) and the Beta Actin (Bio-Rad Laboratories, California, US) primary antibodies were 1:100. Next day, the antibody solution was aspirated, and cells were washed three times with PBS for 5 min. For secondary antibody alone controls, cells were incubated in blocks without primary antibody. All cells were subsequently incubated in secondary antibody Alexa Fluor 488 Phalloidin (Applied Biosystems ThermoFisher, Pleasanton, CA) (prepared as for primary) at a dilution of 1:500 for 1 h at room temperature with gentle agitation. Cells were then washed three times with PBS for 5 min. The coverslips were then removed from the 12-well plate and excess liquid was gently dabbed off with tissue paper. A few drops of 4′,6-diamidino-2-phenylindole (DAPI) were applied to each slide, and the coverslip was gently dropped downwards at a 45° angle. The coverslips were the sealed using nail varnish. The slides were protected from light and stored at 4 °C overnight allowing the nail varnish to dry. Fluorescent images were obtained using a Zeiss Axioimager M2 microscope fitted with an Axiocam 503 imaging device using Zeiss ZEN software (Zeiss Microscopy × 40 magnification). Cell fluorescence was visualised using an EC Plan-Neofluar 63× (1.25) oil objective. The following channels were used for various fluorophores—DAPI (Ex. − 353, Em. − 465), and Alexa Fluor 488 (Ex. − 493, Em. − 517). The ZenBlue (Zeiss) software was used for image acquisition and subsequent analysis.

### Statistical analysis

GraphPad Prism 9.4.1 software (GraphPad Software, San Diego, USA) was used for statistical analysis and graphical representations. Two-way ANOVA followed by Tukey’s multiple comparisons test was used for three or more groups. A p-value of < 0.05 was considered statistically significant.

### Supplementary Information

Supplementary Table 1.

Supplementary Table 2.

Supplementary Table 3.

## Data Availability

The datasets generated and/or analysed during the current study are available upon reasonable request. Researchers interested in accessing the data can contact the corresponding authors.
