# Supplementary material for: SV2B/miR-34a/miR-128 axis as prognostic biomarker in glioblastoma multiforme
Source: Sci Rep. 2024 Mar 19;14:6647. doi: 10.1038/s41598-024-55917-6 (PMC10951322; doi:10.1038/s41598-024-55917-6)
Supplement: Supplementary file 1 — Supplementary Table 1. [file 41598_2024_55917_MOESM1_ESM.docx]

Supplementary Table 1. Common miRNAs (26) in "miRSystem ", "TargetScan", "miRWalk" and "ENCORI" found to target *SV2B*.

| hsa-miR-136-5p |
| --- |
| hsa-miR-143-3p |
| hsa-miR-150-5p |
| hsa-miR-186-5p |
| hsa-miR-371a-5p |
| hsa-miR-495-3p |
| hsa-miR-520d-5p |
| hsa-miR-524-5p |
| hsa-miR-556-3p |
| hsa-miR-9-5p |
| hsa-miR-182-5p |
| hsa-miR-205-5p |
| hsa-miR-210-3p |
| hsa-miR-216b-5p |
| hsa-miR-27a-3p |
| hsa-miR-27b-3p |
| hsa-miR-324-5p |
| hsa-miR-330-3p |
| hsa-miR-34a-5p |
| hsa-miR-362-5p |
| hsa-miR-449a |
| hsa-miR-506-3p |
| hsa-miR-513a-5p |
| hsa-miR-532-3p |
| hsa-miR-96-5p |
| hsa-miR-1269a |
